# Supplementary material for: Complexome profiling of the Chlamydomonas psb28 mutant reveals TEF5 as an early PSII assembly factor
Source: Plant Cell. 2025 Mar 17;37(6):koaf055. doi: 10.1093/plcell/koaf055 (PMC12164590; doi:10.1093/plcell/koaf055)
Supplement: koaf055_Supplementary_Data [file koaf055_supplementary_data.zip › psb28_interactive.zip!/plots/intensity/qualityPlots/peptides/PIK1_Cre05.g245550.t1.1.html]

### Cre05.g245550.t1.1

Trivial name: PIK1  
SeqCoverage [median]: 1.70  
Mass: 237.85 kDa  
  
 

| Sequence | MissedCleavage | Mass [Da] | wtIntensity | muIntensity | Score |
| --- | --- | --- | --- | --- | --- |
| LLIEAASEPR | 0 | 1097.61 | 0.00 | 448.82 | 84.21 |
| VEQRPPAPLPPLTR | 1 | 1569.90 | 0.00 | 390.28 | 65.56 |
|  |  |  | 0.0 | 839.1 |  |
